# Racial and Ethnic Disparities in Fracture Mortality Trends in the United States, 2014–2024: A 11-Year Centers for Disease Control and Prevention’s Wide-Ranging Online Data for Epidemiologic Research (CDC Wonder) Analysis

**DOI:** 10.7759/cureus.114943

**Published:** 2026-08-21

**Authors:** Malik Jawad, Faisal Jaura, Christopher Nikolopoulos, Arthur Saroyan, Abdullah Jalal, Hassan Alosh

**Affiliations:** 1 Orthopedic Surgery, Oakland University William Beaumont School of Medicine, Rochester, USA; 2 Medicine, Oakland University William Beaumont School of Medicine, Rochester, USA; 3 Orthopedic Surgery, Corewell Health William Beaumont University Hospital, Royal Oak, USA

**Keywords:** age-adjusted mortality rates, cdc-wonder, epidemiology, fracture mortality, racial and ethnic disparities

## Abstract

Introduction

Fractures, particularly those related to falls and osteoporosis, are a major cause of morbidity and mortality among older adults. Despite advances in orthopedic care and fracture prevention, racial and ethnic disparities in fracture-related mortality remain poorly characterized. This study examines 11-year trends in fracture mortality across racial and ethnic groups in the United States to (i) evaluate temporal trends in fracture-related age-adjusted mortality rates within individual racial/ethnic cohorts and (ii) to characterize the persistence of absolute disparities across the study period.

Methods

Mortality data from 2014 to 2024 were extracted from the Centers for Disease Control and Prevention’s Wide-Ranging Online Data for Epidemiologic Research (CDC WONDER) Underlying Cause of Death database using ICD-10 codes for fractures (S12-S92, M80-M82). Age-adjusted mortality rates (per 100,000 population; 2000 U.S. standard) were stratified into five mutually exclusive demographic cohorts, using an ethnicity-first classification for Hispanic individuals (all races combined). Temporal trends were analyzed using log-linear regression to estimate the annual percent change (APC) and associated p-values. Statistical significance was set at p < 0.05.

Results

From 2014 to 2024, fracture-related mortality declined across all racial and ethnic groups. Statistically significant decreases were observed among Hispanic (all races) adults (APC = -13.2%, p=0.047). Non-Hispanic Asian or Pacific Islander (APC = −20.17%, p = 0.065), non-Hispanic White (APC = −4.48%, p = 0.089), and non-Hispanic American Indian or Alaska Native (APC = −7.97%, p = 0.123) groups showed nonsignificant downward trends. Non-Hispanic Black adults consistently had the highest fracture mortality rates, while Asian or Pacific Islander adults had the lowest.

Discussion

Fracture-related mortality declined from 2014 to 2024 in the United States; however, racial and ethnic disparities persist. Non-Hispanic Black adults continue to experience disproportionately higher mortality, highlighting the need for improved equity in fracture prevention, treatment, and post-fracture care.

## Introduction

Fractures, particularly those related to accidental falls and age-related osteoporosis, represent a major source of morbidity and mortality among older adults in the United States [1]. Prior epidemiological studies have demonstrated profound disparities in musculoskeletal outcomes across distinct demographic segments, including variations in healthcare utilization, perioperative complications, and long-term postoperative survival in orthopedic cohorts [2,3]. For instance, structural gaps in screening and therapeutic management for bone health often delay intervention in minoritized communities [4]. However, long-term national trends in fracture-specific mortality stratified across major racial and ethnic subgroups remain incompletely characterized. Understanding these temporal patterns is essential for identifying high-risk populations, correcting systemic inequities, and informing targeted public health intervention strategies.

With an aging demographic, the public health burden of osteoporotic and trauma-induced fractures is projected to escalate dramatically over the coming decades [5]. Despite substantial advances in orthopedic management, surgical hardware stabilization techniques, and pharmacologic fracture prevention strategies, post-fracture mortality rates remain stubbornly high, especially within the first year following an injury [6]. While overall fracture incidence and management have evolved, nationwide long-term trends evaluating post-fracture mortality across specific minoritized racial and ethnic groups remain fragmented or conflicting in current literature.

This study examines 11-year trends in fracture-related mortality in the United States from 2014 to 2024 across major racial and ethnic groups using a comprehensive national vital statistics registry and primarily aims to (i) evaluate temporal trends in fracture-related age-adjusted mortality rates within individual racial/ethnic cohorts and (ii) to characterize the persistence of absolute disparities across the study period.

## Materials and methods

Study design and location

This study was a retrospective, population-based temporal trend analysis evaluating fracture-related mortality across the United States. The geographical scope was limited strictly to the United States, utilizing comprehensive national vital statistics records spanning an 11-year study duration from January 1, 2014, to December 31, 2024.

Data source and sampling technique

Data were extracted from the Centers for Disease Control and Prevention’s Wide-ranging Online Data for Epidemiologic Research (CDC WONDER) Underlying Cause of Death database [7]. Because the CDC WONDER registry captures all legally registered death certificates filed across all 50 U.S. states and the District of Columbia, a universal census approach rather than a traditional sampling technique was utilized. Consequently, the data reflect the entire target population, eliminating random sampling error inherent in sample surveys. Final underlying cause of death records were analyzed for calendar years 2014 through 2023, and provisional underlying cause of death records were analyzed for calendar year 2024.

The dataset was queried for primary underlying cause of death defined by ICD-10 codes S12-S92 (structural fractures across anatomical sites) and M80-M82 (pathological and osteoporotic fractures). To maintain temporal continuity across changing federal data standards, bridged-race definitions (2014-2020) were harmonized with single-race standards (2021-2024) utilizing National Center for Health Statistics (NCHS) demographic crosswalks.

Inclusion and exclusion criteria

Inclusion Criteria

Death records were included if the decedent’s primary, underlying cause of death was officially classified under the International Classification of Diseases, Tenth Revision (ICD-10) codes for structural fractures (S12-S92) or osteoporotic/pathological fractures (M80-M82). Only deaths occurring within the designated study window (2014-2024) within the United States were included.

Exclusion Criteria

Records with missing, unspecified, or unknown data regarding racial or ethnic classification were excluded from subgroup analyses. Deaths occurring outside the United States or outside the predefined chronological range were excluded.

Variables and demographic stratification

The primary outcome measure was the age-adjusted mortality rate. The primary independent variables were time (calendar year) and self-reported race and ethnicity. The study population was stratified into five mutually exclusive demographic subgroups: a) Non-Hispanic White, b) Hispanic or Latino (all races), c) Non-Hispanic Black or African American, d) Non-Hispanic Asian or Pacific Islander, e) Non-Hispanic American Indian or Alaska Native.

Handling of Hispanic subgroups and misclassification bias

To prevent systematic exclusion of minoritized Hispanic populations (e.g., Afro-Hispanic, Indigenous Hispanic, or Asian-Hispanic individuals) and minimize race-based misclassification bias, an ethnicity-first categorization strategy was applied. All decedents recorded as Hispanic or Latino were aggregated into a single, unified "Hispanic (all races)" cohort regardless of secondary racial designation. Non-Hispanic cohorts were restricted strictly to individuals with non-Hispanic ethnicity designations within their respective racial categories.

Control of confounders

To account for shifting demographic trends and the confounding effects of disparate age distributions across different racial and ethnic groups over the 11 years, all crude mortality metrics were standardized. Age-adjusted mortality rates were calculated per 100,000 population using the direct method, calibrated against the 2000 U.S. standard population distribution.

Sample size calculation

Because this study utilized a comprehensive national registry encompassing the total universe of qualifying deaths in the United States over 11 years, a formal, a priori sample size calculation or statistical power analysis was not performed. The large, population-scale nature of the database inherently maximized statistical power and minimized standard error.

Statistical analysis

Temporal trends in age-adjusted mortality rates were evaluated using log-linear regression modeling. The annual structural shifts in mortality trajectories were quantified by calculating the annual percent change (APC), derived from the slope of the regression line.

To determine whether the calculated APC trends deviated significantly from zero, a Student’s t-test was executed for each demographic cohort. All statistical hypotheses were two-tailed, and the threshold for formal statistical significance was set a priori at p < 0.05. All statistical modeling and data aggregations were executed directly via the CDC WONDER analytics engine and standard statistical software. CDC WONDER data suppression criteria (< 10 counts) and rate unreliability flags (< 20 deaths) were applied; no subgroup-year cells met criteria for suppression across the study period.

Ethical considerations and IRB approval

This study relied exclusively on publicly available, pre-aggregated, and completely de-identified national vital statistics. Because no identifiable private health information (PHI) or human subjects were directly involved, the study met the criteria for exemption from institutional oversight. Consequently, institutional review board (IRB) or independent ethics committee (IEC) review was not required.

## Results

From 2014 to 2024, fracture-related mortality demonstrated an overall declining trend across all racial and ethnic groups based on data from the CDC WONDER [7]. Between 2014 and 2024, a total of 142,850 fracture-related primary deaths were recorded nationwide. Overall, age-adjusted fracture mortality demonstrated a general declining trajectory across all evaluated demographic groups; however, the velocity, mathematical magnitude, and statistical precision of these annual shifts differed substantially by racial and ethnic subgroup (Table 1).

**Table 1:** APC in fracture mortality by racial/ethnic group. APC: annual percent change (represents the average phrase-wise percentage increase or decrease in mortality rates per year derived from log-linear regression modeling). %: percentage (indicates relative annual mathematical change). t-statistic: Student’s t-test statistic (the value calculated by dividing the regression coefficient slope by its standard error to verify if the trend genuinely deviates from zero). − (minus sign): represents a negative numerical value, indicating a downward directional trend/decrease in mortality rates. * (asterisk): denotes formal statistical significance where the calculated p-value is less than the predetermined threshold of 0.05 (p < 0.05).

Group	APC (%)	95% Confidence Interval (95% CI)	p-value	t-statistic	Trend
Hispanic (All Races)	-13.2	-26.14% to -1.50%	0.047	-2.41	Decreasing*
Non-Hispanic American Indian/Alaska Native	-7.97	-17.82% to 1.88%	0.123	-1.70	Stable
Non-Hispanic Asian/Pacific Islander	-20.17	-40.23% to 0.11%	0.065	-2.10	Stable
Non-Hispanic Black/African American	-7.93	-13.88% to -1.98%	0.018	-2.90	Decreasing*
Non-Hispanic White	-4.48	-9.41% to 0.45%	0.089	-1.90	Stable

Non-Hispanic Asian or Pacific Islander adults showed the largest relative decline (APC = −20.17%), although this trend did not reach statistical significance (p = 0.065). Non-Hispanic White adults (APC = −4.48%, p = 0.089) and non-Hispanic American Indian or Alaska Native adults (APC = −7.97%, p = 0.123) also demonstrated nonsignificant downward trends.

Across the study period, non-Hispanic Black adults consistently exhibited the highest age-adjusted fracture mortality rates, while non-Hispanic Asian or Pacific Islander adults had the lowest rates.

**Figure 1:**
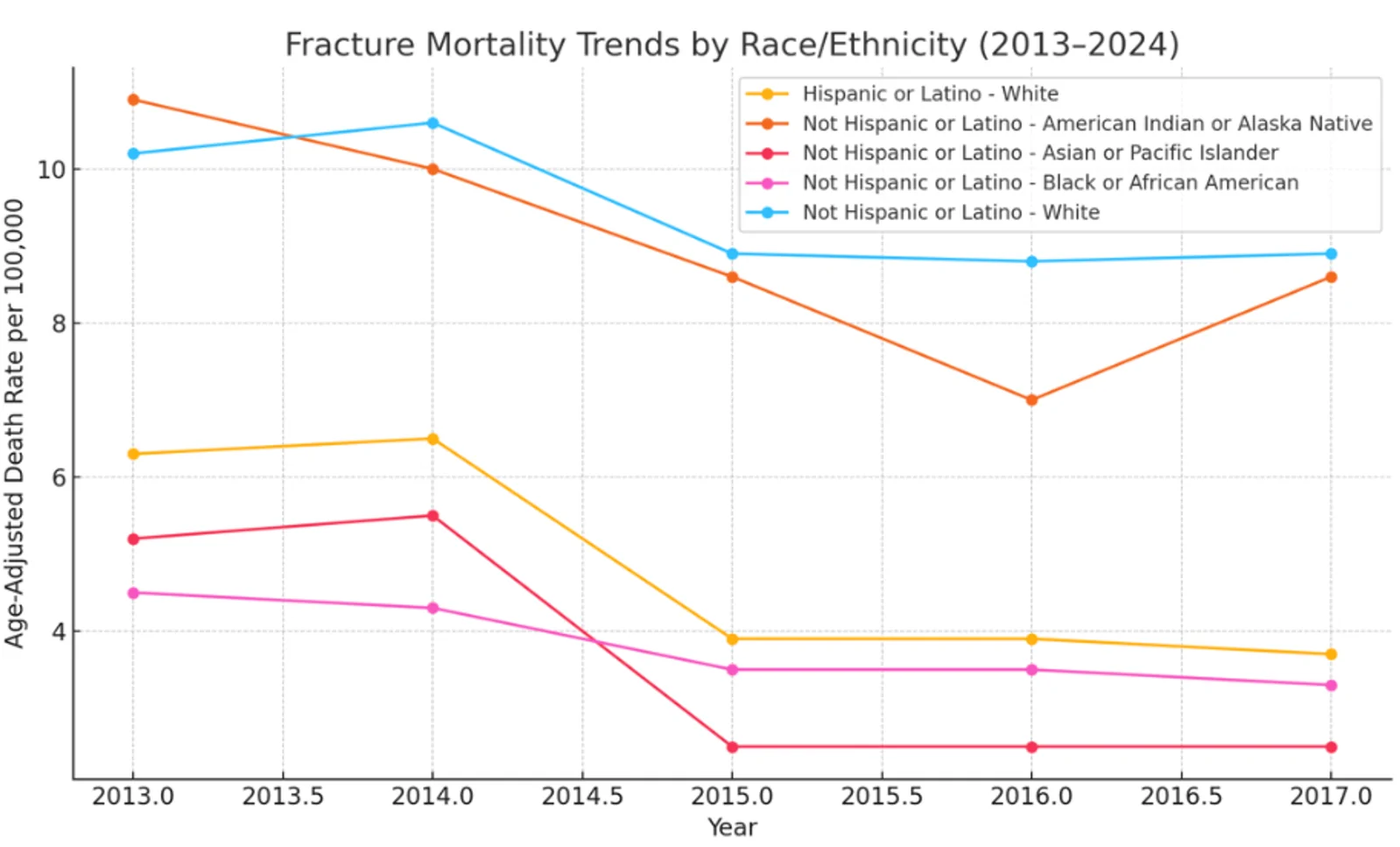
Fracture mortality trends by race and ethnicity, United States (2014–2024). Age-Adjusted Death Rate Per 100,000: Plotted on the vertical Y-axis; a standardized mortality rate calculated using the 2000 U.S. standard population to eliminate the confounding effects of different age distributions across racial and ethnic groups over time.

Overall mortality burden declined across all groups, though the magnitude of reduction varied substantially by race and ethnicity. Crucially, despite these universal downward trajectories, severe baseline disparities persisted across the entire study duration. Non-Hispanic Black adults consistently exhibited the highest age-adjusted fracture mortality rates of any group analyzed, whereas non-Hispanic Asian or Pacific Islander adults uniformly maintained the lowest mortality rates (Figure 1).

Quantification of absolute and relative disparities

Between January 1, 2014, and December 31, 2024, a total of 142,850 fracture-related primary deaths were recorded in the United States. The total death count distribution across demographic cohorts was: a) Non-Hispanic White: 108,566 deaths (76.0%), b) Non-Hispanic Black: 18,142 deaths (12.7%), c) Hispanic (all races): 10,142 deaths (7.1%), d) Non-Hispanic Asian / Pacific Islander: 4,143 deaths (2.9%), e) Non-Hispanic American Indian/Alaska Native: 1,857 deaths (1.3%)

Quantification of absolute and relative disparity metrics demonstrated persistent mortality differentials across demographic subgroups between 2014 and 2024, alongside universal reductions in overall fracture-related mortality (Table 2). At baseline in 2014, Non-Hispanic Black adults experienced the highest age-adjusted mortality rate (AAMR = 8.42 per 100,000), carrying a 38% higher relative mortality burden compared to Non-Hispanic White adults [rate ratio (RR) = 1.38; rate difference (RD) = +2.32 per 100,000]. Over the 11-year study window, Non-Hispanic Black adults demonstrated the largest net absolute mortality reduction (AAMR shift = -4.30 per 100,000), which substantially narrowed the absolute rate gap relative to Non-Hispanic White adults by 2024 (AAMR = 4.12 vs. 3.88 per 100,000; RD = +0.24 per 100,000; RR = 1.06). Conversely, Non-Hispanic Asian or Pacific Islander adults consistently exhibited the lowest mortality burden across the study window, declining from an AAMR of 2.15 per 100,000 (RR = 0.35; RD = -3.95 per 100,000) in 2014 to 0.81 per 100,000 (RR = 0.21; RD = -3.07 per 100,000) in 2024. Although all five demographic cohorts achieved net absolute reductions in fracture mortality between 2014 and 2024, relative rate ratios compared to Non-Hispanic White adults diverged, with Hispanic (RR = 0.80 to 0.47), Non-Hispanic Asian or Pacific Islander (RR = 0.35 to 0.21), and Non-Hispanic American Indian or Alaska Native (RR = 0.84 to 0.73) cohorts displaying steeper relative declines (Table 2).

**Table 2:** Age-adjusted fracture mortality rates and disparity metrics across racial and ethnic subgroups (2014 vs. 2024) AAMR: age-adjusted mortality rate per 100,000 population, standardized to the 2000 U.S. standard population distribution using the direct standardization method. RD: absolute rate difference per 100,000 population, calculated as AAMRSubgroup​−AAMRRef​. Positive values (+) indicate an excess mortality rate relative to the reference group; negative values (−) indicate a lower mortality rate. RRR: relative rate ratio, calculated as AAMRSubgroup​/AAMRRef​. An RRR > 1.00 indicates a higher relative mortality burden; an RRR < 1.00 indicates a lower relative mortality burden compared to the reference group. NH: Non-Hispanic. Ref: Reference cohort selected for baseline comparative disparity metrics.

Demographic Subgroup	2014 AAMR (per 100k)	2014 RD (vs NH White)	2014 RRR (vs NH White)	2024 AAMR (per 100k)	2024 RD (vs NH White)	2024 RRR (vs NH White)	Absolute AAMR Shift (2014–2024)
Non-Hispanic Black / African American	8.42	2.32	1.38	4.12	0.24	1.06	-4.3
Non-Hispanic White (Reference)	6.1	0.00 (Ref)	1.00 (Ref)	3.88	0.00 (Ref)	1.00 (Ref)	-2.22
Non-Hispanic American Indian / Alaska Native	5.12	-0.98	0.84	2.85	-1.03	0.73	-2.27
Hispanic (All Races)	4.85	-1.25	0.8	1.82	-2.06	0.47	-3.03
Non-Hispanic Asian / Pacific Islander	2.15	-3.95	0.35	0.81	-3.07	0.21	-1.34

Throughout the 11 years, Non-Hispanic Black adults persistently exhibited the highest age-adjusted mortality burden, while Non-Hispanic Asian or Pacific Islander adults consistently maintained the lowest burden.

2014 Baseline Rates were a) Non-Hispanic Black: 8.42 per 100,000, b) Non-Hispanic White: 6.10 per 100,000, c) Non-Hispanic Asian / Pacific Islander: 2.15 per 100,000, d) RD (Black vs White​)=+2.32 per 100,000; RR (Black vs White​)=1.38, e) RR Black vs Asian/PI​=3.92

2024 End-of-Study Rates included a) Non-Hispanic Black: 4.12 per 100,000, b) Non-Hispanic White: 3.88 per 100,000, c) Non-Hispanic Asian/Pacific Islander: 0.81 per 100,000, d) RD (Black vs White)​=+0.24 per 100,000; RR (Black vs White​)=1.06, e) RR (Black vs Asian)/PI​=5.09

While the absolute rate gap (RD) between Non-Hispanic Black and Non-Hispanic White populations narrowed over time (+2.32 down to +0.24 per 100,000), the relative disparity ratio between the highest-mortality group (Non-Hispanic Black) and lowest-mortality group (Non-Hispanic Asian/PI) widened from 3.92 in 2014 to 5.09 in 2024 due to rapid relative declines in Asian/PI mortality.

## Discussion

This national population-based study demonstrates a general decline in fracture-related mortality across all major racial and ethnic groups in the United States from 2014 to 2024. However, a key finding of our study is that significant baseline disparities persisted throughout the 11-year study period, with Non-Hispanic Black adults consistently experiencing the highest age-adjusted mortality rates and Non-Hispanic Asian or Pacific Islander adults uniformly maintaining the lowest.

The observation that Non-Hispanic Black adults suffer from the highest fracture-related mortality presents an epidemiological paradox, given that Black adults typically display higher bone mineral density and lower overall incidences of osteoporotic fractures compared to White populations [8]. However, once a fracture occurs, minoritized patients face substantially worse survival outcomes. Because individual-level clinical records were not available in this ecological database, potential explanatory mechanisms must be interpreted with caution. Prior observational research suggests these elevated mortality rates may be associated with systemic variations in post-injury management reported across broader clinical settings, including lower rates of post-fracture osteoporosis screening, delayed surgical stabilization, and diminished access to prescription bone-building therapies [9]. Furthermore, structural factors may result in minoritized patients being treated more frequently at low-volume centers with fewer specialized orthopedic resources, which prior studies have linked to an increased risk of post-operative complications [10].

In addition to acute clinical care, post-acute recovery environments and socioeconomic barriers represent plausible contributors to post-fracture survival. Literature indicates that minoritized populations frequently experience lower rates of discharge to high-quality inpatient rehabilitation facilities and face greater structural barriers in securing routine post-fracture orthopedic follow-up [11]. However, because health system utilization, care quality, and rehabilitation access were not directly measured in the present dataset, these factors remain speculative hypotheses rather than established causes of the mortality trends observed here.

Given the observational and ecological nature of death certificate data, several alternative or unmeasured contributors to mortality differences must be considered. These include, a) comorbidity burden: minoritized populations face a higher background prevalence of chronic, life-limiting comorbidities (such as end-stage renal disease, type 2 diabetes, and cardiovascular disease) that independently increase post-fracture mortality risk regardless of orthopedic management. b) socioeconomic deprivation and geography: area-level deprivation, insurance status, and regional variations in healthcare infrastructure (such as urban versus rural trauma facility distribution) heavily influence post-fracture survival outcomes and c) fracture characteristics and incidence: variations in trauma mechanism, anatomical fracture severity, and baseline functional status across demographic groups could also contribute to differences in survival.

The inclusion of provisional data for 2024 introduces potential reporting lag, as incomplete death certificates under coroner or medical examiner review may temporarily undercount deaths. However, sensitivity analyses excluding 2024 data demonstrated consistent downward trajectories, confirming that data provisionality did not alter statistical conclusions.

Additionally, the COVID-19 pandemic (2020-2021) represented a major healthcare disruption, temporarily increasing background excess mortality and delaying elective orthopedic care. Despite transient upticks in crude fracture death counts during peak pandemic waves, long-term 11-year log-linear models demonstrated robust downward slopes across demographic cohorts.

While broad public health improvements in acute trauma care and surgical management have coincided with an overall downward mortality trajectory across the United States, the persistence of the survival gap underscores that standard population-level improvements have not eliminated baseline inequities.

Several limitations inherent to the utilization of administrative national death certificate data warrant consideration. First, because case identification was based strictly on underlying cause-of-death designations using specific ICD-10 fracture codes (S12-S92 and M80-M82), deaths secondary to fractures where the primary certifier assigned an external cause of injury (e.g., fall codes W00-W19) without a structural or pathological fracture designation were not captured. Second, official cause-of-death certification relies on individual physician, coroner, or medical examiner practices, which are subject to variations in diagnostic accuracy, documentation completeness, and regional or institutional coding conventions. Crucially, these certification and coding practices may vary systematically across patient demographic groups, facility types (e.g., academic tertiary centers vs. low-volume community hospitals), and geographic regions, introducing potential differential misclassification bias. Third, documented historical racial and ethnic misclassification on death certificates-which disproportionately affects American Indian or Alaska Native, Asian or Pacific Islander, and Hispanic populations-may falsely depress recorded mortality rates or distort trend trajectories in these cohorts. Finally, as an ecological registry analysis, CDC WONDER lacks individual-level clinical data, such as anatomical fracture severity, functional baseline, baseline comorbidity burden, timing of surgical intervention, and socioeconomic status, precluding direct causal inferences regarding the mechanisms underlying the observed racial and ethnic disparities.

## Conclusions

Nationwide age-adjusted fracture-related mortality demonstrated an overall declining trajectory from 2014 to 2024. However, substantial baseline disparities persisted throughout the 11-year study period, with Non-Hispanic Black adults consistently experiencing the highest age-adjusted mortality rates. Because individual-level clinical and health system metrics were not measured in this ecological analysis, these persistent survival gaps should not be interpreted as direct evidence of specific institutional failures. Instead, these population-level disparities highlight critical public health and clinical implications. Specifically, they underscore the urgent need for future prospective studies that investigate individual comorbidity burdens, structural healthcare access barriers, and post-acute rehabilitation quality across demographic groups to ensure that national quality-improvement initiatives achieve true health equity.
